# Refining animal welfare of wild boar (*Sus scrofa*) corral-style traps through behavioral and pathological investigations

**DOI:** 10.1371/journal.pone.0303458

**Published:** 2024-05-21

**Authors:** Katharina M. Westhoff, André Fetzer, Zarah Schwan, Kathrin Büttner, Johannes Lang, Michael Lierz

**Affiliations:** 1 Clinic for Birds, Reptiles, Amphibians and Fish, Faculty of Veterinary Medicine, Justus Liebig University Giessen, Hesse, Germany; 2 Unit for Biomathematics and Data Processing, Faculty of Veterinary Medicine, Justus Liebig University Giessen, Hesse, Germany; Universitat Autonoma de Barcelona, SPAIN

## Abstract

Wild boar trapping has been used as a management tool to control wild boar populations. However, it is increasingly criticized due to animal welfare concerns. While cortisol levels have been used to assess trap-related stress in wild boar, data on trap-related injuries and behavioral data are scarce. We aimed to evaluate three different corral-style traps for wild boar according to available mammal trapping standards to investigate and refine animal welfare in wild boar trapping. We examined 138 wild boars captured and killed by head shot in 27 capture events. Traps were closed by remote control only if the complete group were trapped. The behavior of the animals in the trap and during culling was recorded on video. All wild boars were examined and a pathological and radiological examination of the heads for trap- and shot-related injuries followed. Trap-related injuries occurred in 33% of the animals with superficial mild skin defects to skull fractures. One out of three traps met all the set requirements. A wire-meshed trapping system failed all. After installing an incomplete barrier in the center of the trap to slow down trapped animals, the fracture rate in one trap type was significantly reduced by 29% (p < 0.05). Our data showed that the type of trap (*p* = 0.007) and the number of animals trapped at once (*p* = 0.002) had a significant influence on the number of escape attempts. Trapping larger groups reduced the escape attempts. We emphasize the importance of an accurate pathological examination to evaluate animal welfare in traps and call for adjusting the injury categories listed in the standards and make a proposal for wild boar live trapping.

## Introduction

Wild boar (*Sus scrofa*) populations are increasing and expanding not only in Europe, but worldwide, which leads to human-wild boar interactions and conflicts [1, 2]. The presence of wild boar in urban and peri-urban areas, the increasing number of wildlife-vehicle collisions, damage to arable and forestry crops and the risk of wildlife-livestock interactions and disease transmission are numerous reasons for the necessity of a population control [2–4]. However, the high dispersal ability of wild boar makes population control of this species even more challenging [5]. Recreational hunting is the standard approach for wild boar management in Europe [6, 7], but with the spread of African swine fever across Europe since 2014, wild boar trapping received renewed attention as management tool in disease prevention and control [8–10].

Wild boar live traps are used for hunting and culling, especially in peri-urban or urban areas, for translocation and in research [11–13]. Various types of wild boar live traps have been used, ranging from small cage traps over semi-permanent corral-style traps, net traps to permanent corrals [12, 14, 15]. With corral-style traps it is more likely to trap entire sounders, but construction and relocation can be time-consuming [16]. Box traps are easily to move from one area to another, whereas corral-style traps have been shown to be more efficient than box traps in terms of effort, time and money spent [16].

However, wild boar live trapping and subsequent culling is criticized and debated because of animal welfare concerns [17, 18]. From an animal welfare and hunting ethics perspective, it is crucial to assess the impact of the trapping method on the animal’s condition objectively. The live-capture of wild animals has various effects on their welfare and health, leading to behavioral, physical and physiological alterations [15, 19–21]. In the case of wild boar live-capture for translocation or research long-term negative effects also play a role [22].

Physiological alterations such as increased levels of stress hormone or their metabolites, elevated body temperature and blood pressure or changes in hematological variables has been used for animal welfare evaluation in trapping [13, 20, 23, 24]. The capture of free-ranging wildlife can cause a stress response [25]. As the animal experiences a loss of control due to the inability to escape, this might lead to a psychological stress. In addition, a prolonged stay in the trap can lead to an increased stress level as the animal cannot perform its natural behaviors or only to a limited extent [20].

Trap-related physical injuries have been reported in wild boar [14, 26–28] and other wildlife [18, 29]. After the trap gate closes, injuries such as lacerations and abrasions of the facial skin and fractures of the nasal bone can occur as the wild boars charge against the trap walls or attempt to jump up to escape [26]. The trap design and especially the mesh size of wire mesh traps has an effect on the occurrence of injuries [26, 28]. Even in the absence of injuries, capture-related stress may be present, as evidenced by changes in behavior [14]. Behaviors like resting, foraging, escape attempts or behavioral alterations like increased activity can thus be used for the assessment of animal welfare [14]. Excessive escape behavior in particular leads to injury and thus indicates capture-related stress. Therefore, combining pathological examinations with behavioral analyses and physiological parameters is crucial for an animal welfare assessment [18, 24, 30].

The Agreement on International Humane Trapping Standards (AIHTS [30]) require that the welfare of the trapped animal be evaluated based on physiology, behavior, and injuries. Under the terms of this agreement, signatory parties are required to certify restraining and killing traps for the capture of the listed furbearers, whose furs are to be traded among the parties, in accordance with the standards [30, 31]. Although this binding agreement provides international standards for trapping, wild boar is not listed as a target species. Additionally, the International Organization for Standardization (ISO) has published mammal trapping standards for the testing of restraining and killing traps, which target all mammal species, but are not legally binding [32, 33]. Both standards (ISO and AIHTS) established an animal welfare standard, based on certification protocols, which are supposed to be met by each trap type before their widespread use. In case of restraining traps, the ISO testing protocol is mainly based on a pathological evaluation trough numerical scoring of injuries by severity and adjusting a trauma class [33]. In the AIHTS standards, the pathological evaluations are based on a list of behavioral and physical indicators for poor welfare. A restraining trap meets the AIHTS requirements, if at least 80% out of 20 trapped animals of the target species do not show any of the listed indicators [30]. However, especially AIHTS standards are strongly criticized by wildlife researchers and conservationists for being ineffective in ensuring animal welfare due to insufficient and outdated standards and test protocols, for example an incomplete species list, and insufficient thresholds of acceptance [34, 35]. Subsequently, Proulx et. al. [35] proposed new mammal trapping standards with higher acceptance thresholds and an adjusted injury-scoring system.

The increasing use of wild boar traps, especially in Europe, requires a precise animal welfare evaluation of each trap type. For example, in Germany wild boar trapping is restricted by the government and requires a permit, but there are no legal requirements for implementation or trap design [36]. Various wild boar traps with subsequent culling as management tool or hunting method are used without any pretesting of the traps by animal welfare standards. According to the 3R principles (Replacement, Reduction and Refinement), it is necessary to test and improve each trap type in order to minimize stress and trap-related injuries [37]. In Sweden, field testing is mandatory for the certification of most new live traps for wildlife [14].

Besides drop-nets, recently studied by Conejero et al. [15], corral-style traps are the most commonly used trap types for wild boar. Fahlman et al. [14] lately evaluated a smaller wire mesh corral-style trap. They found behavioral changes indicative of trap-induced stress in animals without injuries, making the inclusion of behavioral data essential for evaluating live trapping in wild boar [14]. Individually trapped wild boar exhibited more escape behavior and more escape attempts in the presence of humans than at the beginning of trap closure [14]. Westhoff et al. [24] confirmed this using cortisol levels, showing that wild boars captured in groups had lower cortisol levels than those captured individually. In addition, cortisol levels were higher in wild boar corral-style traps compared to other hunting methods [24]. According to Westhoff et al. [24], reliance on cortisol analysis alone is insufficient to assess the welfare of wild boars in corral-type traps. Therefore, it is crucial to integrate additional data from behavioral analyses and pathological examinations.

The aim of the study was to evaluate three different corral-style traps for wild boar in the field according to available mammal trapping standards and to evaluate the methods to assess animal welfare in traps for wild boar. We focused on two commercially available larger corral-style trap types and one selfmade trap type, which is regularly used in Germany. The capture requirements were to capture the entire group of boars at once and to close the capture gate in real time to prevent exclusion of group members or injury from the closing gate. This required video monitoring and live remote gate closure. We addressed the following questions: 1) Does trapping with these corral-style trap types meet the requirements of the available trapping standards? 2) Are there differences in the trap types in terms of animal welfare? 3) What measures can be derived to improve animal welfare in wild boar trapping?

## Materials and methods

### Study area

Wild boar trapping was conducted during the regular hunting season at 3 sites in Hesse (central Germany) from May 2019 to August 2021. Site 1 was a 22,000-ha state-owned forest (51.0° N/8.8° E) with an average annual precipitation of 774 mm and an average annual temperature of 8.6°C [38]. The altitude ranged from 260 to 698 m above sea level. Except from wild boar the ungulate community consisted mainly of red deer (*Cervus elaphus*) and roe deer (*Capreolus capreolus*).

Site 2 was an abandoned 250-ha fenced military site (49.9° N/8.8° E) with 80% forest area and 20% open land, not accessible to the public. An average annual precipitation was 798 mm, average annual maximum daily temperature 12.6°C and altitude 139 m above sea level [38]. The only other wild ungulates at this site were roe deer. In May 2020 European bison (*Bison bonasus*) and in spring 2021 Przewalski horses (*Equus przewalskii*) were located to the site. Since then, electric fences protected the wild boar traps to keep the bisons and horses at a distance. Site 3 was an active 2,250-ha military training ground with public access (50.9° N/9.4° E) with 50% forest area and 50% grassland. The average annual precipitation was 623 mm, average annual maximum daily temperature 13.8°C and altitude 273 m above sea level [38]. The other wild ungulates at this site were roe and red deer.

The forest area of all three sites mainly consisted of mixed broad-leafed forest with primarily European beech (*Fagus sylvatica*), European spruce (*Picea abies)*, European oak (*Quercus robur*), Douglas fir (*Pseudotsuga menziesii*) and pine (*Pinus pinea*). In a European context, these forests served as important habitats for wild boar and supported significant wild boar populations. Recent data from Greiser et. al. [39] indicate a hunting yield of over 3 wild boars per square meter in the past years. The management of wild boar hunting at all three sites was entrusted to the personnel of the respective forest services, who collaborated with private hunters. At site 2 hunting has been restricted to 10% of the complete area since 2019.

### Trapping procedure

The use of three different types of corral-style traps set up by the local hunting authorities to capture wild boar as described in Westhoff et al. [24] was monitored. All trap types were designed for mobile use and for capture of entire sounders. The *Krefelder* trap type (Krefelder Vario, Thomas Vennekel & Georg Achten GBR, Krefeld, Germany) was made of 2 m high wooden plank walls between metal support, set up in a round shape with a floor size of 60 m^2^ (9 m diameter) and 2 opposite drop gates. The *Selfmade* trap type was made of 2 m high wooden plank walls with metal support from the outside, set up in rectangular shape (5 x 10 m) with 2 opposite drop gates and planks to cover the 90° corners. The third trap type *JagerPro* (M.I.N.E.® Trap System, Jager Pro Inc., Fortson GA, USA) consist of mesh wired wall elements of 1.80 m height, set up in round shape of 90 m^2^ (10.7 m diameter) with one drop gate. The mesh size was 5 x 15 cm up to a height of 25 cm and 10 x 25 cm above. We set up 2 *Krefelder* traps, 2 *Selfmade* traps and 2 *JagerPro* traps at site 1, 1 *Krefelder* trap and 1 *Selfmade* trap at site 2 and 2 *Krefelder* traps at site 3.

The staff of the local forest services baited the traps with corn following local restrictions. The Hessian Hunting Law limits bait feeding on native grain, corn and peas to a maximum of one liter per day and feeding site [40]. Wildlife cameras were used to monitor whether trap sites were visited by wild boars. All traps were set up with 3 wildlife cameras (RevierSpion 4.0 G, RevierSpion LTE 4G, GF Trading GmbH, Havixbeck, Germany; X-view Wildkamera 6.5 G, X-view Wildkameras, Dietfurt, Germany) covering the area around the gates and the inside. When wild boars appeared at the trapping site, additional LTE video live cameras (Reolink Go, Reolink, Hongkong, China) were fixed, or a WIFI network (4G router, Case "Elite", Wildlife Technology Concepts GmbH, Frankfurt, Germany) was installed at the trapping site to run WIFI cameras (Reolink Argus 2, Reolink, Hongkong, China). Traps were set during daylight hours to prepare for trapping events after dawn, when wild boars would enter the trap with all their sound members. Wildlife cameras informed a person not on site about entering animals and the person triggered the gate via GSM (Global System for Mobile Communications), radio trigger or WIFI (Case "Elite", Wildlife Technology Concepts GmbH, Frankfurt, Germany), while watching the animals inside the trap via live video-recording. During trap testing, the triggering technology needed to evolve (from radio to local WIFI) to provide real-time remote triggering, thus being able to trap entire sounders and to reduce the risk of injuries from the dropping gates. The time from gate closure until arrival of the culling team was noted, and culling was documented for all capture events with full video documentation (see *Behavioral observations*).

Trained hunters killed wild boars inside the trap by headshot (rifle with .22 lr caliber) from an elevated wooden stand. Two battery-powered LED construction spotlights illuminated the entire trap area, allowing the shooter to keep track of the animals for better targeting and placement of follow-up shots if necessary. The time of each shot was documented. In total, 10 traps (*Krefelder*: *n* = 5; *JagerPro*: *n* = 2; *Selfmade*: *n* = 3) were used from October 2019 to August 2021, and 139 wild boars were caught and 138 killed in 27 trapping events (S1 Table). One wild boar escaped after capture and will not be mentioned further below.

After evaluating the video footage from the first captures, it was suspected that the length of the trap room of the *Selfmade* trap type could pose a problem in terms of nose injuries, when running against the gates directly after gate closure. Therefore, in July and August 2020 (since capture event 14), barriers consisting of round wooden posts driven into the ground were installed in the center of the trap room to slow down the animals. Due to the results during the study, we excluded the *JagerPro* trap from the study from May 2021 onwards. (See *Results*).

### Behavioral observations

A video camera was installed on a pole at a height of 3.5 m to record the entire trap area and the gates. Motion-triggered cameras were used, which stopped recording after movement of the animals stopped and started recording with new movement. The ethogram of Fahlman et al. [14] was adapted to include seven behavioral categories (comfort behavior, still, metabolic behavior, locomotion, security behavior, escape, intraspecific aggression) with multiple behaviors [41]. An unbiased observer conducted the behavioral observation afterwards. Only trapping events with complete video recording of the gate closure, the intermediate time, and the time until the last animal was shot were included in the analysis. Due to the stop of the video recordings, when wild boar were inactive, individual observation was not possible and the average group behavior was analyzed (according to Fahlman et al. [14]). The observer classified the behavior in each video sequence into the behavioral categories using the ethogram and noted the duration. The different behaviors were summed for the overall behavioral category [41]. In this analysis, we focus on escape behavior from the ethogram data set. Here the total trap time was defined as the time between gate closure and the last shot and divided it into three periods: *gate closure* (gate closure till 5 min afterwards), *intermediate time* and *human arrival* (shooters arrival till last shot) (S2 Table). We summed the behavior *charging against the walls* and *crashing against the gate* as *escape attempts* for each trapping event and divided it by the number of wild boars per capture event.

### Carcass examinations

**Fig 1:**
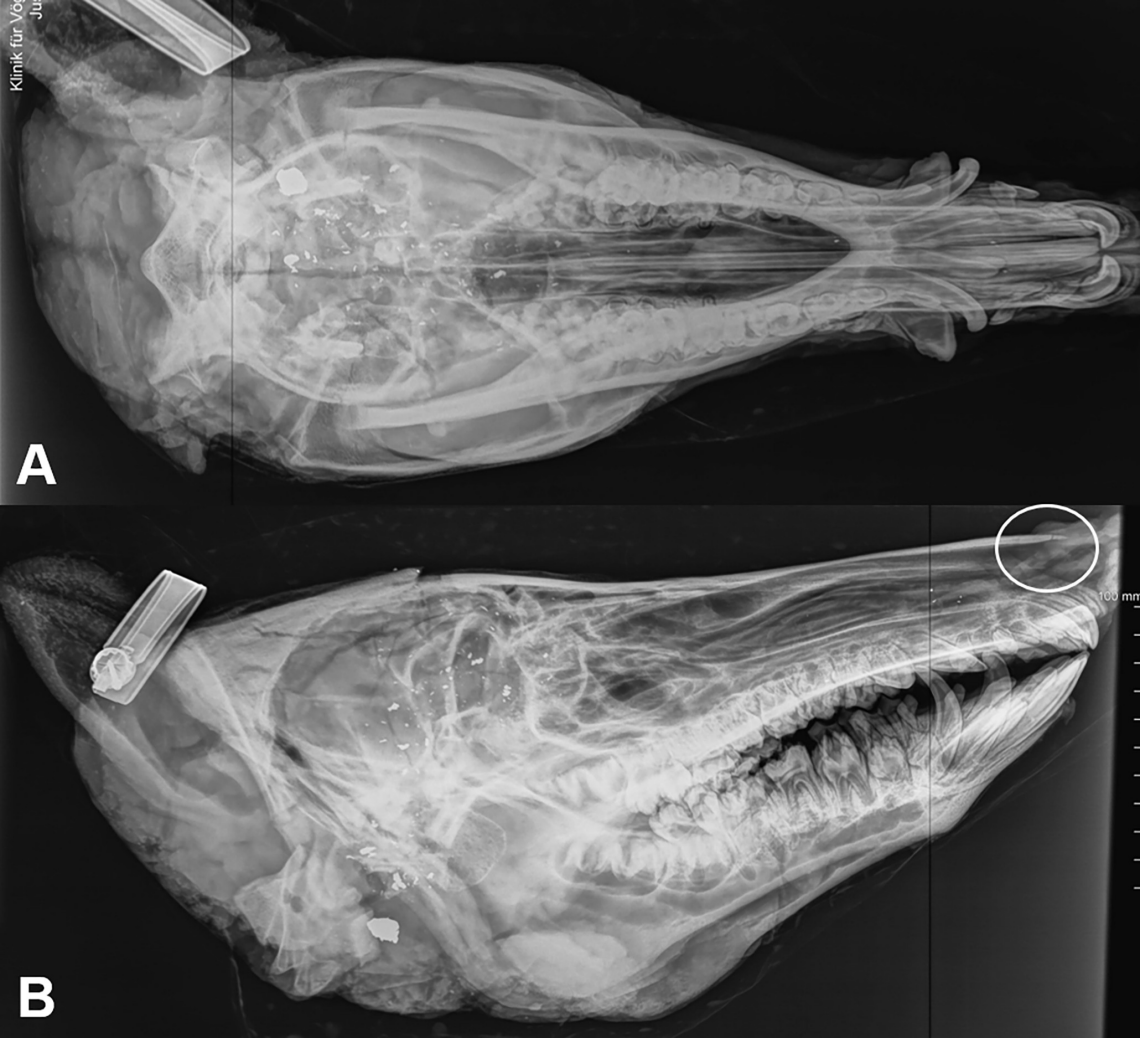
X-rays of wild boar heads caught in corral-style traps and killed with headshots. **(**A) Dorsoventral x-ray of the head with bullet and bullet particles associated with multiple skull fracture lines. (B) Laterolateral x-ray with multiple skull fracture lines in association with bullet particles and bullet near condyles. White ring marks nasal bone tip fracture line (medium size head: 68 kV, 4.14 mA).

After all animals were shot, the trap was entered and a veterinarian confirmed the death of the animals. All carcasses were individually marked with ear tags in the order of shooting. Each animal underwent examination for shot- and trap-related injuries, sex and age. Age groups (juveniles < 12 months, yearlings = 12–23 months or adults ≥ 24 months) were determined according to Güldenpfennig et al. [42] based on dental age estimation. After evisceration, the carcass weight without viscera was determined. The heads of all wild boars were cut off directly on site and temporarily stored refrigerated (+4° C) or frozen (-20° C) at the Clinic for Birds, Reptiles, Amphibians and Fish. There, laterolateral and dorsoventral x-rays of the heads were taken (HF400VA x-ray tube Gierth X-Ray international GmbH, Riesa, Germany; DX-S X-ray developer AGFA Healthcare, Bonn, Germany, settings for medium size head: 68 kV, 4.14 mA). X-rays were used to locate bullets and bullet particles, to evaluate gunshot-related findings, and to detect fractures in conjunction with the pathological examinations. The x-ray examination for diagnosing fractures was compared with the findings from the pathological examinations. A veterinarian then performed a pathological examination of all the heads, including skinning. All pathological findings directly related to gunshot wounds, bullets or bullet particles were classified as shot-related injuries, but are not discussed further here (Fig 1A). All other findings not related to shooting or trapping (e.g., ectoparasites, abscesses, old wounds) were documented but will not be discussed here. Trap related injuries were documented and assessed according to AIHTS [30] (Table 1), ISO-10990-5 [33] (Table 2 and S3 Table) and Proulx et. al. [35] (S2 Table). Pathological findings were grouped into superficial and deep, defined as within the skin or extending into the subcutaneous tissue or musculature (S2 Table). Tooth tip fractures without exposure of the pulp cavity were documented. However, they were not scored because they are not listed in any of the above standards (Table 1). Although some authors only count the occurrence of one type of injury, here each injury was counted individually, because not only the occurrence and type of injury, but also the number is important for the assessment of animal welfare.

**Table 1:** Distribution of wild boars with indicator injuries according to AIHTS^a^ caught and killed in corral-style traps in 27 capture events.

Number of wild boars	Selfmade		JagerPro	Krefelder	Total
**n = 138**	n = 66		n = 9	n = 63	n = 138
	Without barrier	with barrier			
	n = 31	n = 35			
No indicator injury acc. to AIHTS^a^	20	33	1	53	107
in %	65%	94%	11%	84%	78%
Min. 1 indicator injury acc. to AIHTS^a^	11	2	8	10	31
in %	35%	6%	89%	16%	22%

^a^ Anonymus (1998): Agreement on International Humane Trapping Standards between the European Community, Canada and the Russian Federation. In: Official Journal of the European Communities 42, S. 43–57

**Table 2:** Number of wild boars with trap-related injuries scored according to ISO 10990–5^a^ caught and killed in corral-style traps in 27 capture events.

Number of wild boars	Selfmade		JagerPro n = 9	Krefelder	Total
n = 138	n = 66			n = 63	n = 138
	without barrier	with barrier			
	n = 31	n = 35			
adjusted ISO^b^ trauma class					
mild	1	4	0	5	10
moderate	10	2	0	8	20
moderately severe	0	0	3	3	6
severe	1	0	5	2	8

^a^ ISO 10990–5, 1999: Animal (mammal) traps—Methods for testing restraining traps. Annex C; adjusted for wild boar trapping

Because the available standards are primarily designed to assess the welfare of trapped furbearers, they do not list some injuries, such as skull fractures. Therefore, a strict scoring according to the listed injuries and an adjusted scoring for wild boar were performed (S3 Table). According to the pathological observations listed in Annex C of ISO-10990-5 [33], nasal bone tip fractures were classified as “simple rib fractures” (30 points) and comminuted nasal bone fractures were classified as “comminuted rib fractures” (50 points). For the trauma classification, less than 3 mild injuries were classified as “mild”. In the presence of a fracture with an associated hematoma, the latter was not classified as a separate injury, because a fracture without a hematoma would be considered a *postmortem* change. Despite that, all trap-related hematomas were documented (S3 Table). According to the listed injuries in Proulx et. al. [35], nasal bone tip fractures were classified with 30 or 50 points (depending on the severity) and comminuted nasal bone fractures with 100 points. The cumulative injury score was the sum of an animal’s various scores, adjusted for whether these multiple injuries would have a compounding effect on the welfare or survival of the released animals [35]. Again, fractures and their associated hematoma were scored as one unit, as described above.

### Statistical analysis

To analyze the behavioral data, it was first calculated whether there was a relationship between the proportion of escape attempts and total trapping time using a Spearman correlation. In addition, the relationship between the number of escape attempts and the group size was investigated using Spearman correlation. For this the relative escape attempts (escape attempts total /number of wild boars caught) and the number of captured animals was used (S2 Table).

An analysis of covariance was used for the relative proportion of escape attempts (escape attempts total/number of wild boars caught). The relative proportion of escape attempts served as the dependent variable, trap type (*Krefelder*, *Selfmade*, *JagerPro*) as the independent variable, and number of animals captured as the covariate (S2 Table).

To determine whether radiographic examination is a useful method for detecting nasal fractures in this setting, specificity and sensitivity were calculated using a classic four-field table. The number of positive radiographic findings (nasal fracture) was contrasted with the number of fractures detected at necropsy.

A two-tailed Exact Fisher’s test was performed to test whether the number of trap-related fractures in the *Selfmade* trap type was related to the presence of a barrier. All statistics were performed using SAS 9.4 [43].

#### Ethics statement

All described sampling methods were conducted during or after normal legal hunting activities due to the laws of the Federal Republic of Germany and the Federal State of Hesse. No animals were harmed or killed for our sampling specifically. Therefore, it was a non-experimental study in the sense of the German Animal Welfare Act and approval by an ethics committee was not required.

## Results

### Animals trapped

Overall, we examined 138 wild boars caught in 27 capture events, 62 males, 76 females, ranging from juveniles (<12 months) to adults (≥ 24 months) and from 2.2 kg to 65 kg carcass weight (without viscera) (S1 Table). The sounder size varied from 1 to 20 ($\bar{x}$ = 5.1) animals per capture event (S1 Table).

### Behavioral observations

Sufficient video material was available to evaluate wild boar behavior for 18 of the 27 captures (capture ID 3–6, 7, 9, 11,12, 14, 16–19, 21, 22, 24, 26, 27; *n* = 106). Due to mainly technical problems, there was not enough video material for the remaining captures. In total, 9 h and 16 min of video were collected. The average video length per capture was 31 min 56 sec. This data set contains trapping events from 3 *JagerPro*, 6 *Krefelder* and 9 *Selfmade* trap types. Group size ranged in total from 1–20 ($\bar{x}$ = 5.89), in *JagerPro* trap from 1–6 ($\bar{x}$ = 2.67), in *Krefelder* trap from 1–20 ($\bar{x}$ = 8.17) and in *Selfmade* trap from 4–8 animals ($\bar{x}$ = 5.44).

**Fig 2:**
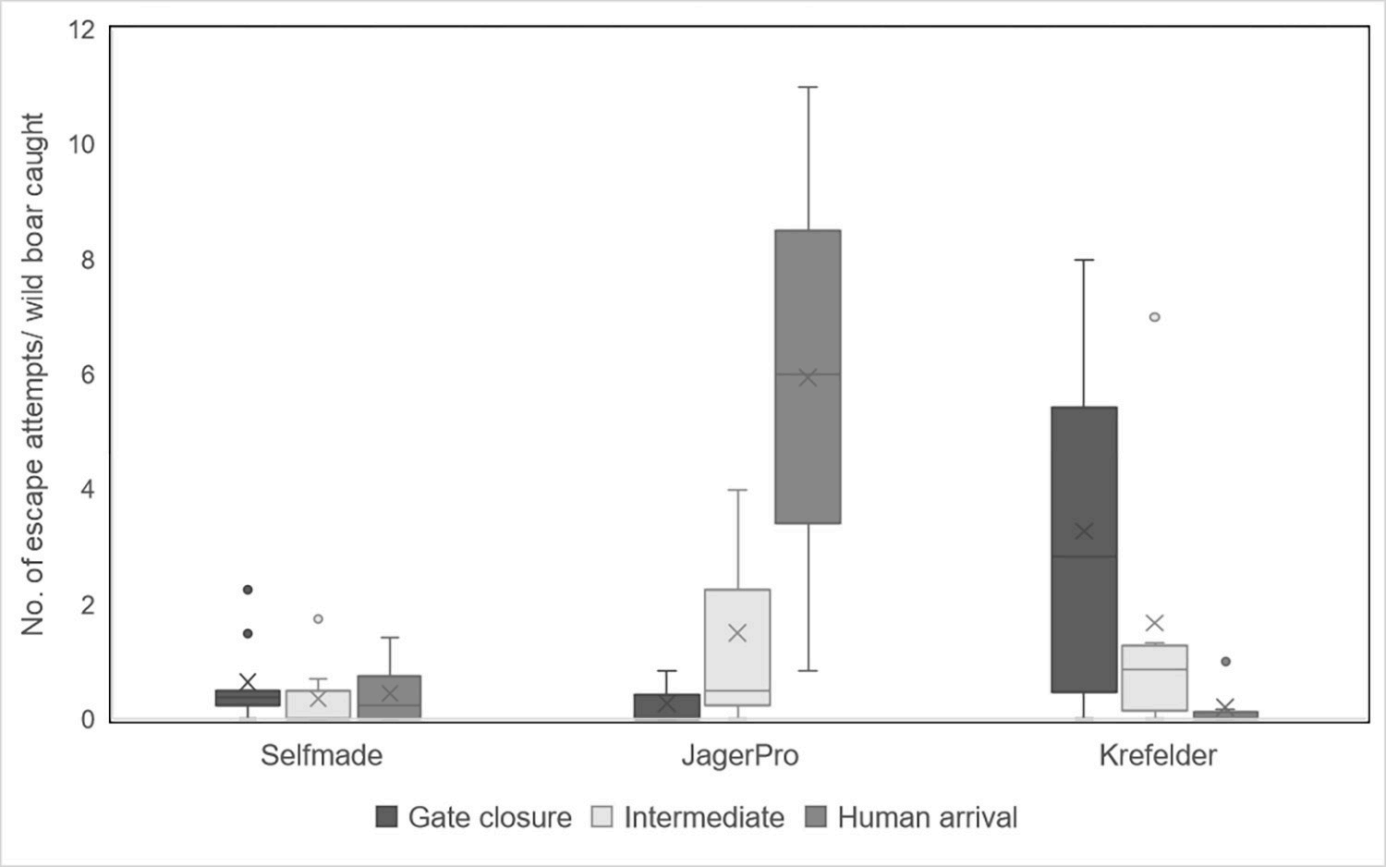
Number of escape attempts per captured wild boars separated by time period (gate closure, intermediate, human arrival) and trap type (*Selfmade*, *JagerPro*, *Krefelder*). 138 wild boars caught with corral-style traps in 18 capture events.

Fig 2 shows the descriptive statistics of the number of escape attempts per captured wild boar (relative escape attempts) during different time periods. Descriptively, the relative number of escape attempts increased over time and peaked at the arrival of the shooter in the *JagerPro* trap (*gate closure*: $\bar{x}$ = 0.28, *SD* = 0.48; *intermediate*: $\bar{x}$ = 1.50, *SD* = 2.18; *human arrival*: $\bar{x}$ = 5.94, *SD* = 5.08). In the *Krefelder* trap, the relative number of escape attempts decreased over time. Relatively more escape attempts per captured wild boar were observed during gate closure and fewer during human arrival (*gate closure*: $\bar{x}$ = 3.27, *SD* = 3.28; *intermediate*: $\bar{x}$ = 1.68, *SD* = 2.67; *human arrival*: $\bar{x}$ = 0.20, *SD* = 0.40). Wild boars in *Selfmade* trap type made relatively fewer escape attempts during the intermediate time period. The number of escape attempts per captured wild boar was relatively similar across the different time periods than for the other trap types (*gate closure*: $\bar{x}$ = 0.65, *SD* = 0.73; *intermediate*: $\bar{x}$ = 0.36, *SD* = 0.58; *human arrival*: $\bar{x}$ = 0.45, *SD* = 0.57; Fig 2).

The total time in the trap (gate closure to last shot) varied from 38–137 min ($\bar{x}$ = 86 min) among the 18 captures. No significant correlation was found between the proportion of escape attempts and the total trapping time (*r*(16) = -0.10, *p* = 0.689). Therefore, trapping time was not included in further calculations.

**Fig 3:**
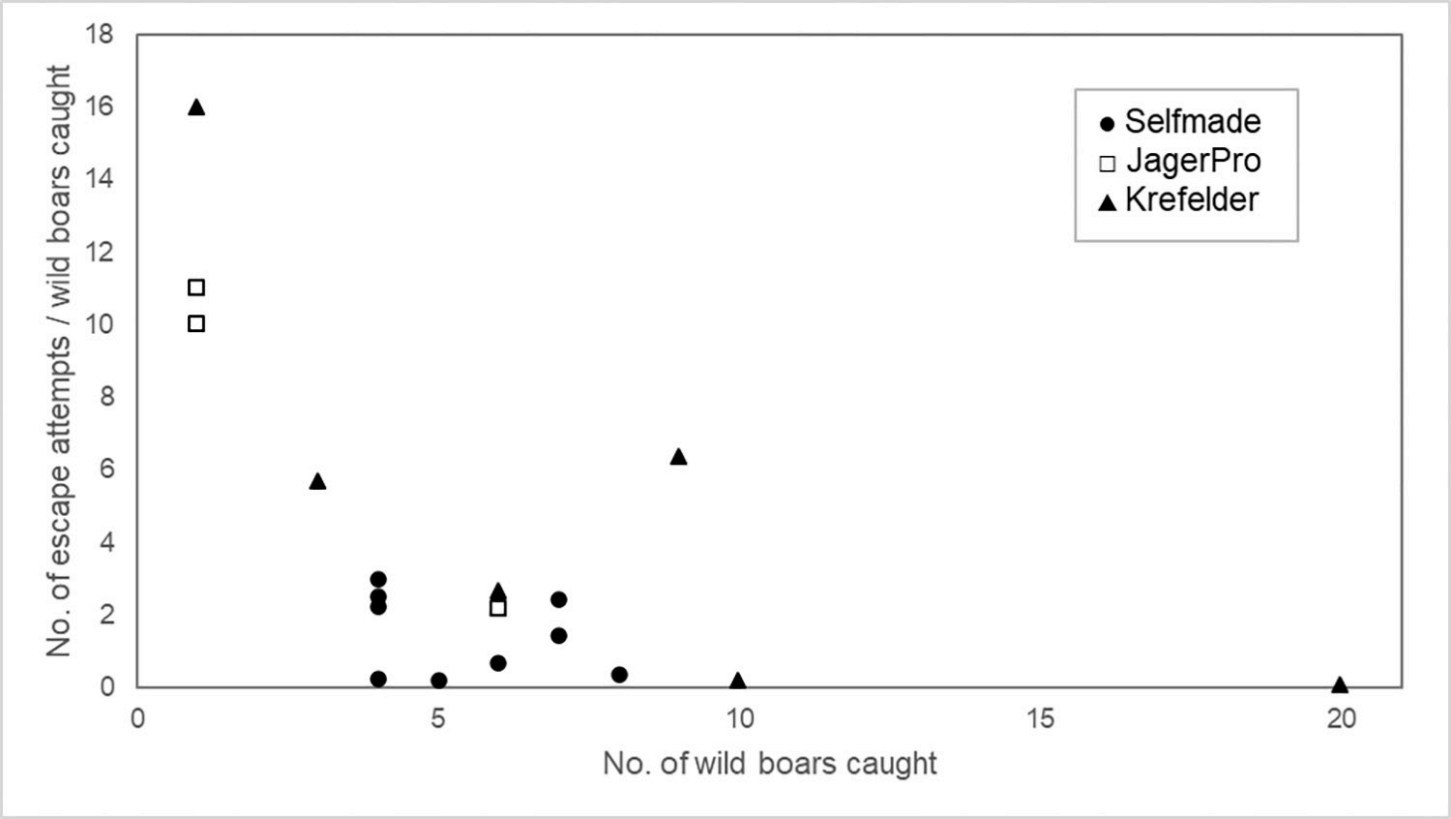
The number of escape attempts per captured wild boars in relation to the number of captured wild boar. 138 wild boars caught with corral-style traps in 18 capture events. Different symbols mark the trap type (*Selfmade*, *JagerPro*, *Krefelder*).

In addition, the relationship between the number of escape attempts and group size was investigated (Fig 3). There was a significant negative correlation between the number of escape attempts per captured wild boar and the number of captured animals (*r*(16) = -0.65, *p* = 0.003).

The type of trap (*F* = 7.15, *df* = 2, *p* = 0.007) and the number of trapped animals (*F* = 14.13, *df* = 1, *p* = 0.002) had a significant influence on the number of escape attempts. If the number of trapped animals increased by one, the number of escape attempts decreased by 0.67 (and vice versa). The highest number of escape attempts per captured wild boar in total was found in the *JagerPro* trap ($\bar{x}$ = 7.72, *SD* = 4.84) followed by the *Krefelder* ($\bar{x}$ = 5.15, *SD* = 5.93) and the *Selfmade* ($\bar{x}$ = 1.46, *SD* = 1.11). However, a significant difference was only found between the *Krefelder* and *Selfmade* trap types in the number of escape attempts (*p* = 0.011). There was no significant difference between *JagerPro* trap and *Krefelder* or *Selfmade* trap type. All other pairwise comparisons were not significant.

### Radiographic examinations of the heads

Radiographic examination revealed fractures in 35 of 138 (26%) wild boars and no fractures in 103 (75%) wild boars.

### Pathological assessment

In the general examination trap-related injuries were diagnosed in 45 out of 138 wild boars (33%; *Selfmade*: *n* = 18/66; *JagerPro*: *n* = 8/9; *Krefelder*: 19/63), primarily in the head region (S2 Table). Superficial skin abrasions, excoriations and lacerations were detected in 12% of the animals (16/138; *Selfmade*: *n* = 2/66; *JagerPro*: *n* = 6/9; *Krefelder*: 8/63). In 9% of the wild boars, deep skin cuts or lacerations were found (12/138; *Selfmade*: *n* = 1/66; *JagerPro*: *n* = 3/9; *Krefelder*: 8/63). One animal (ID 27) had a compound upper jaw fracture with bilateral mandibular fractures, which was detectable by external examination due to a strong axis deviation of the jaw bones.

**Fig 4:**
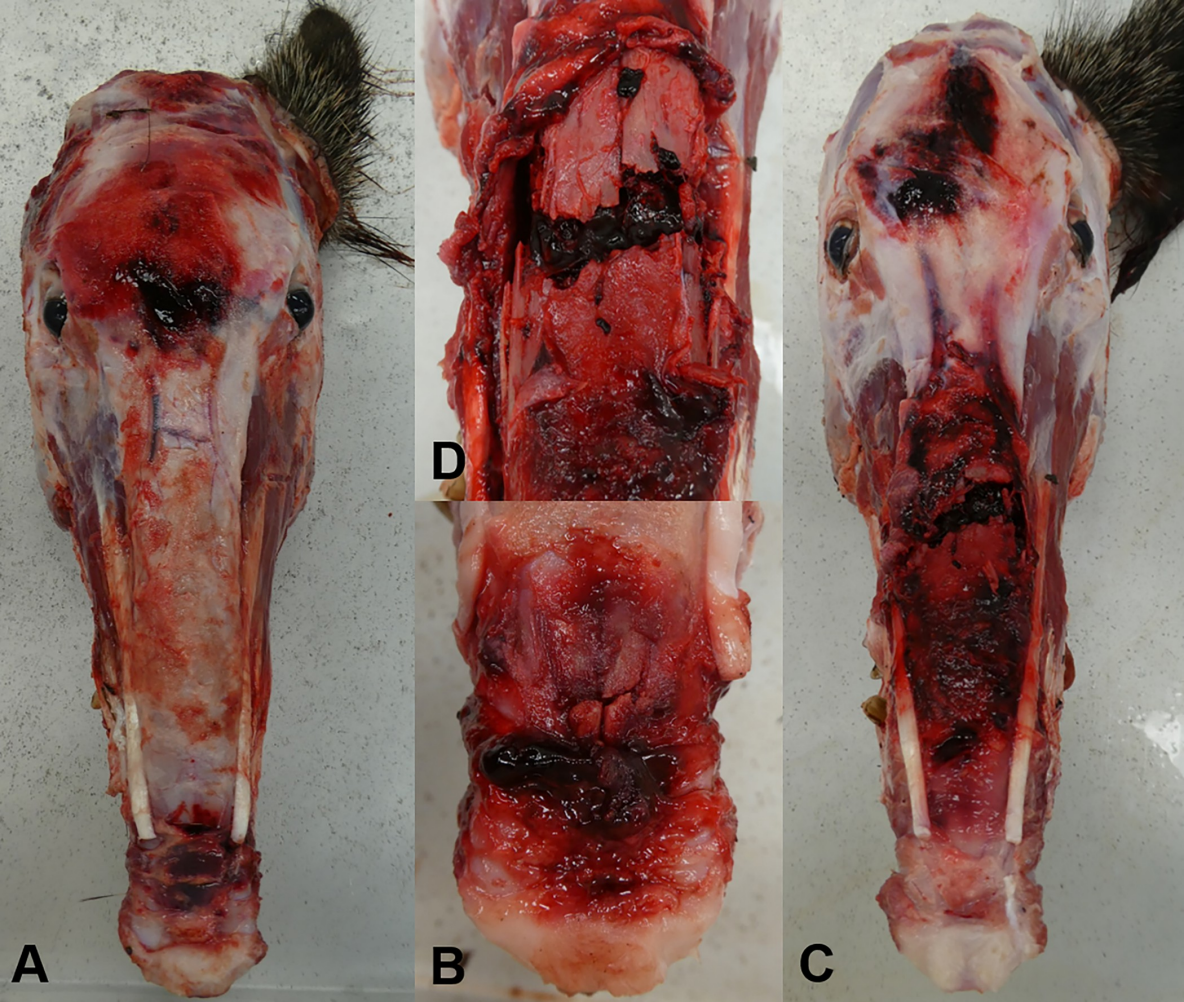
Skinned wild boar heads caught in corral-style traps and killed with headshots. **(**A) Wild boar skull with hematoma in the frontal area between the eyes in direct association with the bullet hole (shot-related injury) and a hematoma at the junction of the nasal bridge to the snout (trap-related injury). (B) Close up of the nasal bone tip fracture after resection of the covering hematoma in A. (C) Wild boar skull with hematoma in the frontal area (shot-related injury) and a severe hematoma and comminuted nasal bone fracture along the nasal bridge (trap-related injury). (D) Close up of C after resection of the covering hematoma.

After skinning the heads, subcutaneous hemorrhage was seen in 40 animals (29%; *Selfmade*: *n* = 18/66; *JagerPro*: *n* = 8/9; *Krefelder*: 14/63). We discovered 30 wild boars with at least one fracture (22%; *Selfmade*: *n* = 13/66; *JagerPro*: *n* = 7/9; *Krefelder*: 10/63). All fractures were closed and located in the head region, mainly at the nasal bone. One specific type of fracture was frequently found: a nasal tip fracture at the junction of the nasal bridge and the snout (Fig 4A and 4B). In addition, multiple comminuted fractures centered on the bridge of the nose involving the os nasale, os incisivum, and partially the maxilla were noted (Fig 4C and 4D, Table 2). In 3 wild boars (ID 27, 43, 78) 2 fractures were found. In 7 wild boars (5%; *Selfmade*: *n* = 0/66; *JagerPro*: *n* = 3/9; *Krefelder*: 4/63), dental findings such as dental abrasions, tooth tip fractures without and tooth fractures with exposure of the pulp cavity were detected.

When comparing the radiographic method with the pathologic examination for the diagnosis of fractures, the radiographic method was found to have a sensitivity of 63% and a specificity of 85%.

The highest proportion of animals with external injuries (superficial and deep skin alterations, dental findings) originated from the *JagerPro* (7 out of 9 caught wild boar in this trap type), the lowest from the *Selfmade* trap (3/66). The trap *JagerPro* had the highest percentage of animals with fractures (*n* = 7/9) all of which were comminuted nasal bone fractures. The highest proportion of nasal tip fractures were detected in *Selfmade* trap type (12/13; *Krefelder*: *n* = 8/10; *JagerPro*: *n* = 0/7) and the highest proportion of more complicated fractures in *JagerPro* trap type (7/7; *Krefelder*: *n* = 2/10; *Selfmade*: *n* = 1/13).

According to AIHTS, at least one indicator injury was found in 31 (22%) of the 138 captured wild boars (Table 1). Of the indicator injuries listed in the AIHTS, fractures and fractures of permanent teeth exposing the pulp cavity were observed. None of the other listed alterations were seen macroscopically. Of the 66 wild boars captured in total in the *Selfmade* trap, 53 (80%) had no indicator injuries and 13 (20%) animals had at least one. Comparing the occurrence of animals with trap-related fractures before (without barrier) and after (with barrier) was reduced from 35% (11/31) to 6% (2/35) (Table 1). There was a significant correlation found between the installation of a barrier and the occurrence of fractures. The number of fractures in the *Selfmade* trap without a barrier was significantly higher than the number with a barrier. (Exact Fisher’s test; two-tailed *p* = 0.004). In the *JagerPro* trap, 8 out of 9 (89%) wild boars had at least one indicator injury and in the *Krefeld*er trap there were 10 (16%). Based on these findings, the *JagerPro* trap was eliminated during the study as early results indicated that this trap would not meet the AIHTS requirements. According to the AIHTS and our data the *Selfmade* trap with barrier and the *Krefelder* trap meet the requirements, but *JagerPro* trap did not.

According to ISO 10990–5 trap-related injuries (Table 1) were scored and the trauma class was adjusted (S3 Table). 10 wild boars were classified with mild trauma (7%; *Selfmade*: *n* = 5/66; *Krefelder*: 5/63), 21 with moderate trauma (15%; *Selfmade*: *n* = 12/66; *Krefelder*: 9/63), 3 with moderately severe (2%; *JagerPro*: *n* = 1/9; *Krefelder*: 2/63) and 10 with severe trauma (7%; *Selfmade*: *n* = 1/66; *JagerPro*: *n* = 7/9; *Krefelder*: 2/63). Thus, most severe injuries were observed in wild boars caught in the *JagerPro* trap.

According to standards proposed by Proulx et. al. [35] “acceptable restraining trap systems are expected, at a 95% confidence level, to hold ≥ 85% of target animals for a specific time period without serious injuries (≤ 50 points), signs of distress or excertion (≤ 50% of the capture time), and significant physiological stress changes”. Using the strict cumulative injury score (no skull fractures or hematomas scored), 2 animals from the *Krefelder* trap (2/63) and one animal from the *JagerPro* trap (1/9) were scored with >50 points. Hence, all trap types except the *JagerPro* trap would meet the requirements. Using our adjusted cumulative injury score (S3 Table), 2 animals from the *Selfmade* trap without barrier (2/31), 0 animals from the *Selfmade* trap with barrier (0/35), 6 animals from the *Krefelder* trap (6/63), and 8 animals from the *JagerPro* trap (8/9) were scored with >50 points. In addition to *JagerPro* trap, the *Krefelde*r trap would just not meet the requirements in this case (84% instead of 85%).

## Discussion

In order to assess animal welfare in wild boar live trapping, we evaluated corral-style traps according to available mammal trapping standards (AIHTS [30], ISO 10990–5 [33], Proulx et. al. [35]). The AIHTS standards have long been criticized by scientists and animal rights activists [34]. Proulx et. al. [35] response to these standards contains adjusted injury categories and a more stringent threshold. However, both standards are based on the capture of single small predatory mammals (furbearers), with an emphasis on limb injuries. The injury categories are adapted to this situation and are only partially applicable to wild boar trapping. This also applies to the injury categories listed in ISO 10990–5 [33]. In the case of trapping wild boar, injuries occur mainly in the area of the head [14, 26], except for wild boar net trapping, where limb injuries also occur more frequently [15].

The way in which injuries are assessed and scored is also important. For example, it makes a large difference, whether the occurrence of the injury category (e.g. minor cutaneous laceration, 5 points) is taken into account [14] or the frequency of the injury (4 minor cutaneous lacerations: 4 x 5 = 20 points). We think that the frequency (multiple injuries of one injury category) should be added together according to Annex C.3 of the ISO 10990–5 [33], because the severity of an injury does not depend solely on the type and location, but also on their number and extent. Regarding the extent of an injury, size thresholds of the injury category would be recommended, as a *deep cut* (30 points, [35]) of 1 cm in length or 30 cm in length would make a difference in the assessment of the severity of the injury. There are several theories in pain research about the modulation of pain stimuli. Some research suggests that pain stimuli may sum when they differ in intensity and spatial extent [44, 45]. Thus, it would make a significant difference to the animal whether it suffers a single injury or multiple injuries in one category. However, there is also a known neurophysiological response called “Diffuse Noxious Inhibitory Control” in which a pain inhibitory effect is triggered by simultaneous stimulation of pain receptors in other parts of the body [46, 47]. In this case, it could be argued that because of this pain-overlapping effect, it does not make much difference to animal suffering whether an animal experiences multiple injuries in one category. Nevertheless, chronic pain and prospects for recovery must also be considered in the assessment of animal suffering, as live traps are also used for catch and release of animals. However, since a trap must be evaluated for all events, the most reasonable scenario should be considered, and therefore the welfare evaluation of live traps should include the frequency of injuries.

In the analysis of the behavioral data, we used escape attempts per number of wild boars captured. However, it is not clear whether one individual or all animals are equally responsible for the number of escape attempts. Although the covariance analysis fits the data well according to the residuals, the results should be interpreted with caution because the distribution of the number of animals captured per trap type is very heterogeneous and there are only 3 capture events in the *JagerPro* trap in the data set. This is due to the early interruption of trapping with the *JagerPro* trap caused by severe injuries.

This study focused on head injuries, as head injuries were expected to be main injuries in corral-type traps [14, 26]. Therefore, only the heads were subjected to detailed pathological examination and radiography. Histopathological examinations were not performed. As a result, myocardial degeneration, spinal cord injury and skeletal muscle degeneration, which are included as injury categories in existing standards, may have been missed. Skeletal muscle degeneration has already been identified in trapped wild boar [15]. Nevertheless, the organs were evaluated while all carcasses were eviscerated directly on site. A rare spinal cord injury would have been detected in the form of neurological symptoms, leg paresis or paralysis. Only mild muscle degeneration would probably have remained undetected.

We evaluated the method of radiological examination of the heads and found a sensitivity of 63% and specificity 85%, which means that 37% of the fractures would not have been detected without subsequent pathological examination and 15% were falsely positive. This applies in particular to nasal tip fractures due to their high proportion. Radiological examination can therefore serve as an additional tool for the detection of fractures, but cannot replace a detailed pathological examination.

In the behavioral analysis, we observed a descriptive increase in the number of escape attempts per wild boar over the different time periods in the *JagerPro* trap, especially after the arrival of the shooter. This is in line with the results of Fahlman et al. [14] for smaller wire mesh traps. The high number of escape attempts in *JagerPro* traps combined with the nature of the injuries (comminuted fractures centered on the nasal bridge) and dental findings, is most likely directly related to the construction of the trap. The animals bite into the metal wire of the trap, causing the long upper jaw and nose to be pushed through the mesh of the trap. If the animal then moves or jumps, this creates a force that is likely to cause the bridge of the nose to break. Mesh size as a cause of trap-related injuries in metal mesh traps has been mentioned earlier [28]. Lavelle et al. [28] found a reduced injury rate in smaller sized mesh compared to wider mesh sized panels and recommended tightly spaced mesh. Regarding the construction, it is important to note that the visual transmission of a wire mesh, as opposed to wooden blanks, is most likely the critical difference that explains the different behavior of the animals and the resulting injuries. Lavelle et al. [28] reported enshrouding of the traps prevented the animals from escaping and reduced movement, which made it easier to shoot the animals.

In contrast, in the *Krefelder* trap, escape attempts appeared to be highest in the early phase after the gate was closed and lowest when the shooters arrived. Statistical analysis showed a significant difference between the *Krefelder* trap and the *Selfmade* trap type in the number of escape attempts. A possible reason for the observed difference in the data set can be attributed to the presence of two outliers (trapping event IDs 21, 26). These two trapping events documented a very high number of escape attempts. No simple explanation is possible here, as the construction of the traps is very similar. Both are closed wooden traps with two drop gates. The outline of the *Krefelder* trap is rounded in contrast to the rectangular shape of the *Selfmade* trap, and the length of the *Selfmade* trap is 1 m longer than the diameter of the *Krefelder* trap. Furthermore, we did not see any obvious differences (age or sex distribution) in these two trapping events that would explain the high number of escape attempts. Other factors, such as stress in the trapped group (e.g. social stress due to rank fights), stress prior to trapping or increased stress due to disturbance around the trap, may be possible explanations.

This study showed that the number of animals trapped had a significant effect on the number of escape attempts per animal. As the number of trapped animals increased, the number of escape attempts per animal decreases. Since escape behavior can lead to injuries, trapping entire sounders is preferable from an animal welfare perspective. These results are in line with previous studies in which the capture of single individuals is associated with higher cortisol levels and increased escape behaviors than wild boar captured in a larger group [14, 24].

Regarding the injury data, the overall injury rate (wild boar with at least one injury) of 33% (45/138) observed in our study fits into the very wide range of 5 to 65% injury rates described in wild boar trapping [12, 14, 15, 26, 28]. However, this numeric value should be treated with caution as it does not provide information on the type and severity of injuries. Therefore, cumulative injury scores as proclaimed in the above-mentioned trapping standards [33, 35] are preferable.

The external injuries documented in our study are most likely related to the animals’ attempts to escape after closing of the gate. The mainly superficial skin defects can be explained by the behavior of the animals after the gate has closed. The animals run against the gate and the side walls or try to lift or jump up on them. The more sensitive and less hairy skin around the nose, mouth, and eyes is primarily affected, and superficial abrasions or lacerations may occur. Superficial abrasions or lacerations is a type of injury regularly described in live capture studies of wild boar [12, 14, 15, 26, 28]. More serious injuries, such as nose fractures, have been the subject of only a few reports [26, 28]. While Fahlman et al. [14] described a nasal bone fissure and Lavelle et al. [28] nasal bone fractures, the present study allows us to demonstrate that a variety of skull fractures can occur during wild boar live trapping. To our knowledge, nasal tip fractures, which were relatively common in this study (*n* = 20 out of 30 animals with fractures), have not been described by other studies. Only skinning and precise dissection of the nasal tissues revealed these fine fracture lines, which are usually obscured by hematoma and easily missed. All but one of the detected fractures (*n* = 30) would have been missed without pathologic examination because they were closed fractures and not visible from the outside of the carcass. Radiographs were helpful in diagnosis of fractures, but were not reliable, especially in detecting nasal tip fractures. Therefore, these types of fractures may have been missed in previous studies.

AIHTS, ISO 10990–5, and Proulx’s new proposal are good reference points for assessing the welfare of wild boar trapping, and the standards provide a foundation. Nevertheless, an adjustment of the injury categories for trapping wild boar is necessary. The assignment of a numerical score to the head injuries identified here (e.g. nasal bone fractures) should also be the subject of further discussion. The nasal bone fractures seen in this study, especially the nasal tip fractures, are not listed in the existing injury categories. Therefore, an assessment of the severity of these injuries needs to be made in the adjustment process. In terms of injury categories, rib fractures may be comparable. Both are known to be painful but often treated conservatively in human patients [48–50]. However, it should be noted that, the wild boar’s nose has an important function as a digging tool for foraging and that the sense of smell is of much greater importance to an animal. We therefore suggest a score of 30 points for nasal tip fractures or fissures and a score of 50 points for nasal tip fractures with extensive hematoma, and a score of 100 points for central and complicated nasal bone or maxillary fractures. Hematomas on the bridge of the nose and snout were also noted frequently in our study. They should not be counted as an additional injury if they occur in conjunction with a fracture, as a fracture without hematoma is considered a postmortem change.

Accordingly, we propose the inclusion of the following injury categories: Subcutaneous snout, chin or nasal bridge hematoma (15 points), major subcutaneous hematoma at the head region (30 points), nasal bone tip fissure or fracture with mild hematoma (30 points), nasal bone tip fracture with severe hematoma (50 points), comminuted nasal bone fracture (100 points), compound upper or lower jaw fracture (100 points).

After reviewing videos of some trapping events and discovering an increasing number of nasal tip fractures in the *Selfmade* trap, we assumed that the rectangular base structure of this trap type with a length of 10 m causes the animals to pick up excessive speed when running towards the gates. This resulted in higher forces onto the nasal bone when crushing onto the gate immediately after closure. Placing a barrier made of round wooden posts in the center of the *Selfmade* trap to reduce the speed of the wild boar running towards the closed gate significantly reduced the number of fractures by 29% **(**11/31 to 2/35; *p* = 0.004). This is a very simple and inexpensive way to refine wild boar trapping with this type of trap in accordance with the 3 R’s principle. At this time, we found less nasal tip fractures in the *Krefelder* than in the *Selfmade* trap, so it was decided not to do this in the *Krefelder* trap. In the end, however, the number of escape attempts in the *Krefelder* trap was higher, especially in the early phase after the gate was closed. Therefore, a barrier in the middle of the trapping area should also be considered for the *Krefelder* trap. It may have a positive effect here as well and may be an explanation for the differences seen in the number of escape attempts when comparing these two trap types.

We found no relation between total trapping time and the number of escape attempts. However, the time spent in the trap should be kept as short as possible to minimize suffering in the event of injuries, especially if this occurred at the start of the trapping process [24, 51]. In the case of closed traps, this is very likely, as we saw the highest number of escape attempts in the early phase. We believe that the nasal fractures result from the first escape attempts after the gate closes.

Trap size may be a factor in the capture of whole sounders. Larger traps may result in increased injury rates due to higher speed when charging against the walls, as shown in this study. On the other hand, smaller traps such as cage traps may be counterproductive if the goal is to capture entire sounders [16]. However, in order to prevent social isolation and its consequences like stress [24] and increased escape behavior shown in this study, this should be the goal from an animal welfare perspective.

In summary, our data demonstrate that a precise pathologic examination is crucial for the detection of trap-related injuries, in particular the nasal bone tip fractures described here. Our data showed that out of the three trap types tested, the *Selfmade* trap met all the requirements of the AIHTS and the new proposal by Proulx et. al. [30, 35]. The *Krefelder* trap met the AIHTS requirements, but only narrowly missed Proulx’s new proposal by 1%, when using an adjusted cumulative injury score. However, the differences between the *Krefelder* and the *Selfmade* traps are difficult to explain and require further investigation. The *JagerPro* trap did not meet any of the above requirements.

Applying the 3Rs principle to the different types of traps, the results of our study show that it is possible to replace the *JagerPro* trap with better types of wild boar traps (replacement) and that is possible to reduce the suffering of wild boar trough appropriate measures. These measures include the construction of a barrier to reduce the speed during escape behavior within a trap, the use of closed trap types instead of wire mesh types and the trapping of groups instead of single animals (refinement).

## Conclusions

Trap-related injuries, especially fractures, hematomas and lacerations at the head and nose, occur to varying degrees in corral-style traps depending on the type of trap and measures to reduce them must be implemented. Barriers to slow down the animals are one simple method represented here. The capture of single wild boars should be avoided and the use of the *JagerPro* trapping system and therefore most likely similar wire mesh wall traps must be rejected due to animal welfare concerns. An adjustment of the injury scoring system for trapping wild boar should be implemented in wildlife trapping standards.

## Supporting information

S1 Table Data from 138 wild boars trapped in 27 capture events with three corral-style trap types (n = 10) in Hesse, Germany, in 2019–2021. (DOCX)

S2 Table Data from video analysis of 18 capture events of 106 wild boars caught and killed in corral-style traps in Hesse, Germany, 2019–2021. (DOCX)

S3 Table Trap-related pathological findings documented in 45 of 138 wild boars caught and killed in corral-style traps in Hesse, Germany, in 2019–2021. (DOCX)
