# Supplementary material for: Refining animal welfare of wild boar (Sus scrofa) corral-style traps through behavioral and pathological investigations
Source: PLoS One. 2024 May 21;19(5):e0303458. doi: 10.1371/journal.pone.0303458 (PMC11108160; doi:10.1371/journal.pone.0303458)
Supplement: S1 Table — (DOCX) [file pone.0303458.s001.docx]

**Supplementary S1Table.** Data from 138 wild boars trapped in 27 capture events with three corral-style trap types (n = 10) in Hesse, Germany, 2019-2021.

| **Capture event** | **Date** | **Site** | **Trap type^a^** | **No. of caught and shot animals** | **Estimated age distribution^b^** | **Time of capture** | **Total time in trap^c^ [h:min]** |
| --- | --- | --- | --- | --- | --- | --- | --- |
| **1** | 22 Oct 2019 | 1 | J | 1 | y | 02:53 | 00:39 |
| **2** | 9 Dec 2019 | 1 | J | 1 | j | 18:37 | 00:43 |
| **3** | 9 Dec 2019 | 1 | S | 6 | j | 23:41 | 00:57 |
| **4** | 15 Feb 2020 | 1 | J | 5 | 3 x y, 2 x j | 21:39 | 01:00 |
| **5** | 5 Mar 2020 | 1 | S | 7 | j | 04:05 | 00:51 |
| **6** | 12 Mar 2020 | 1 | S | 5 | j | 02:05 | 01:09 |
| **7** | 26 Mar 2020 | 2 | S | 4 | y | 21:05 | 00:39 |
| **8** | 14 Apr 2020 | 2 | K | 3 | 2 x y, 1 x j | 22:11 | 00:38 |
| **9** | 15 Apr 2020 | 1 | S | 4 | j | 21:21 | 00:43 |
| **10** | 20 Jun 2020 | 2 | K | 7 | 2 x y, 5 x j | 01:18 | 00:51 |
| **11** | 26 Jun 2020 | 2 | S | 4 | 3 x y,1 x j | 01:16 | 00:55 |
| **12** | 8 Jul 2020 | 1 | J | 1 | j | 22:29 | 00:38 |
| **13** | 15 Jul 2020 | 2 | S | 1 | y | 22:18 | 00:55 |
| **14** | 6 Jan 2021 | 2 | S | 7 | j | 03:20 | 01:43 |
| **15** | 17 Mar 2021 | 2 | K | 2 | j | 00:11 | 01:49 |
| **16** | 8 Apr 2021 | 2 | K | 10 | 2 x a, 8 x j | 00:01 | 01:37 |
| **17** | 14 Apr 2021 | 2 | S | 8 | 1 x a, 7 x j | 00:38 | 01:56 |
| **18** | 25 Apr 2021 | 1 | J | 1 | y | 21:47 | 01:38 |
| **19** | 3 May 2021 | 1 | K | 20 | 2 x a, 2 x y, 16 x j | 23:20 | 02:02 |
| **20** | 17 May 2021 | 1 | S | 1 | a | 02:13 | 01:51 |
| **21** | 26 May 2021 | 3 | K | 3 | 3 x a | 02:34 | 01:39 |
| **22** | 10 Jun 2021 | 3 | K | 6 | 1 x y, 5 x f | 23:50 | 01:43 |
| **23** | 29 Jun 2021 | 2 | K | 2 | 2 x y | 04:10 | 01:31 |
| **24** | 8 Jul 2021 | 3 | K | 1 | a | 01:33 | 01:23 |
| **25** | 8 Jul 2021 | 2 | S | 15 | 1 x y, 14 x f | 21:57 | 01:45 |
| **26** | 6 Au2021 | 3 | K | 9 | 2 x y, 7 x j | 00:40 | 01:59 |
| **27** | 14 Aug 2021 | 2 | S | 4 | 4 x a | 00:21 | 01:52 |

^a^ Estimated age according to Güldenpfennig et al. (2021) (j = juvenile; y = yearling; a = adult)
^b^ J = *JagerPro*, K = *Krefelder*, S = *Selfmade*
^c^ Time from closing the gate to first shot
