# Supplementary material for: Refining animal welfare of wild boar (Sus scrofa) corral-style traps through behavioral and pathological investigations
Source: PLoS One. 2024 May 21;19(5):e0303458. doi: 10.1371/journal.pone.0303458 (PMC11108160; doi:10.1371/journal.pone.0303458)
Supplement: S2 Table — (DOCX) [file pone.0303458.s002.docx]

**Supplementary S2Table.** **Data from video analysis of 18 capture events of 106 wild boars caught and killed in corral-style traps in Hesse, Germany, 2019-2021.**

| **Capture event** | **Trap type^a^** | **No. of animals caught (n)** | **Time period "gate closure"** | | **Time period "intermediate"** | | **Time period "human arrival"** | | **Total** | |
| --- | --- | --- | --- | --- | --- | --- | --- | --- | --- | --- |
|  |  |  | **No. of escape attempts** | **Duration [min]** | **No. of escape attempts** | **Duration [min]** | **No. of escape attempts** | **Duration [min]** | **No. of escape attempts** | **Total trap time^b^ [min]** |
| **4** | J | 6 | 5 | 5 | 3 | 46 | 5 | 12 | 13 | 63 |
| **12** | J | 1 | 0 | 5 | 0 | 28 | 11 | 5 | 11 | 38 |
| **18** | J | 1 | 0 | 5 | 4 | 83 | 6 | 12 | 10 | 100 |
| **16** | K | 10 | 2 | 5 | 0 | 88 | 0 | 11 | 2 | 104 |
| **19** | K | 20 | 0 | 5 | 0 | 105 | 1 | 27 | 1 | 137 |
| **21** | K | 3 | 13 | 5 | 4 | 86 | 0 | 11 | 17 | 102 |
| **22** | K | 6 | 8 | 5 | 7 | 85 | 1 | 26 | 16 | 116 |
| **24** | K | 1 | 8 | 5 | 7 | 71 | 1 | 8 | 16 | 84 |
| **26** | K | 9 | 52 | 5 | 5 | 103 | 0 | 18 | 57 | 126 |
| **3** | S | 6 | 2 | 5 | 0 | 44 | 2 | 9 | 4 | 58 |
| **5** | S | 7 | 0 | 5 | 0 | 40 | 10 | 16 | 10 | 61 |
| **6** | S | 5 | 1 | 5 | 0 | 54 | 0 | 10 | 1 | 69 |
| **7** | S | 4 | 2 | 5 | 7 | 28 | 3 | 7 | 12 | 40 |
| **9** | S | 4 | 1 | 5 | 0 | 27 | 0 | 14 | 1 | 46 |
| **11** | S | 4 | 6 | 5 | 2 | 45 | 1 | 12 | 9 | 62 |
| **14** | S | 7 | 3 | 5 | 5 | 88 | 9 | 14 | 17 | 107 |
| **17** | S | 8 | 3 | 5 | 0 | 102 | 0 | 9 | 3 | 116 |
| **27** | S | 4 | 9 | 5 | 1 | 102 | 0 | 10 | 10 | 117 |

^a^ J = *JagerPro*, K = *Krefelder*, S = *Selfmade*^b^ total trap time = time from trap closure till last shot
