# Supplementary material for: Refining animal welfare of wild boar (Sus scrofa) corral-style traps through behavioral and pathological investigations
Source: PLoS One. 2024 May 21;19(5):e0303458. doi: 10.1371/journal.pone.0303458 (PMC11108160; doi:10.1371/journal.pone.0303458)
Supplement: S3 Table — (DOCX) [file pone.0303458.s003.docx]

**Supplementary S3Table.** **Trap-related pathological findings documented in 45 of 138 wild boars caught and killed in corral-style traps in Hesse, Germany, 2019-2021.**

| **ID** | **Cap-ture event** | **Trap type^a^** | **Sex^b^** | **Carcass mass^c^ [kg]** | **Superficial skin abrasion, excoriation or laceration; location size [cm]** | **Deep skin cut or laceration; localization size [cm]** | **Subcutaneous hemorrhage; location and size [cm]** | **Bone or tooth injuries** | **ISO trauma class^d^** | **Adjusted ISO trauma class^d^** | **Proulx cumulative lesion score^e^** | **Adjusted Proulx cumulative lesion score^e^** |
| --- | --- | --- | --- | --- | --- | --- | --- | --- | --- | --- | --- | --- |
| **1** | 1 | J | m | 54.1 | snout 3 x 1, 1 x 0.5, 2.5 x 0.5, nasal bridge 3 x 1 |  | nasal bridge 15 x 8, chin 6 x 6 | tooth (I1) fracture | severe | severe | 50 | 80 |
| **2** | 2 | J | m | 20 | nasal bridge 2 x 3 |  | nasal bridge 15 x 8 | comminuted nasal bone fracture | mild | moderately severe | 5 | 105 |
| **10** | 4 | J | f | 40.4 | snout 1 x 1, 1 x 1 | snout 4 x 3 | nasal bridge 17 x 6, gum 3 x 3 | comminuted nasal bone fracture | moderately severe | severe | 50 | 155 |
| **11** | 4 | J | f | 45.1 | snout 3 x 1.5, 1 x 0.5 |  | nasal bridge 19 x 7 | comminuted nasal bone fracture | moderate | severe | 5 | 110 |
| **12** | 4 | J | f | 47.9 | nasal bridge 2 x 1, snout 1 x 1.5 | gingiva 2 x 1.5 | nasal bridge 20 x 10, snout circular 5 x 5 | comminuted nasal bone fracture | moderately severe | severe | 50 | 150 |
| **14** | 4 | J | m | 20.4 |  |  | nasal bridge 7 x 5, chin 4 x 3 | comminuted nasal bone fracture | mild | moderately severe | 15 | 115 |
| **22** | 6 | S | m | 27 |  |  | snout circular 2 x 2 | nasal bone tip fracture | mild | moderate | 0 | 30 |
| **26** | 6 | S | m | 24.9 |  |  | nasal bridge 3 x 2.5 | nasal bone tip fracture | mild | moderate | 0 | 30 |
| **27** | 7 | S | f | 29.3 |  | tongue 2 x 0.5 | nasal bridge 10 x 4, snout circular 2 x 2 | Compound upper jaw fracture, bilateral mandibular fracture | moderate | severe | 30 | 245 |
| **28** | 7 | S | f | 30.3 |  |  | nasal bridge 2 x 1 | nasal bone tip fracture | mild | moderate | 0 | 30 |
| **29** | 7 | S | f | 33.2 |  |  | nasal bridge 3 x 2, 6 x 3, 2 x 1 | nasal bone tip fracture | moderate | moderate | 0 | 80 |
| **30** | 7 | S | f | 31.5 |  |  | nasal bridge 2 x 1 |  | mild | mild | 0 | 15 |
| **31** | 8 | K | f | 42.3 | face 2.5 x 0.5 | snout 3.8 x 0.5 | snout 2 x2 |  | moderate | moderate | 30 | 50 |
| **32** | 8 | K | f | 36.5 | snout 1 x 0.5, face 1.5 x 1 |  | snout 2 x 2, nasal bridge 1 x 1 |  | moderate | moderate | 5 | 40 |
| **33** | 8 | K | f | 18.7 |  | snout 3 x 0.5 |  |  | mild | mild | 30 | 30 |
| **37** | 9 | S | f | 19.3 | face 4 x 1 |  | nasal bridge 2.5 x 1.5 | nasal bone tip fracture | mild | moderate | 5 | 35 |
| **39** | 10 | K | f | 16.1 |  |  | nasal bridge 2 x2 | nasal bone tip fracture | mild | moderate | 0 | 30 |
| **41** | 10 | K | f | 22 |  | snout 1.5 x 0.8 |  |  | mild | mild | 30 | 30 |
| **43** | 10 | K | m | 20.4 | tongue 2.5 x 0.5, 1 x 0.5, nasal bridge 2 x 0.5, face 2 x 0.5 | tongue 0.5 x 0.3, snout 2 x 0.5 | nasal bridge 2 x 2 | nasal bone fracture with distal tip fracture | moderately severe | severe | 100 | 230 |
| **45** | 11 | S | m | 37.5 |  |  | snout circular 5 x 2 | nasal bone tip fracture | mild | moderate | 0 | 50 |
| **46** | 11 | S | f | 29.1 |  |  | snout circular 4 x 2 | nasal bone tip fracture | mild | moderate | 0 | 50 |
| **47** | 11 | S | f | 27.9 |  |  | nasal bridge 2 x 1.5 | nasal bone tip fracture | mild | moderate | 0 | 30 |
| **48** | 11 | S | f | 28.5 |  |  | snout and nasal bridge 2 x 3 | nasal bone tip fracture | mild | moderate | 0 | 45 |
| **49** | 12 | J | m | 31.3 |  |  | snout 2 x 2,  nasal bridge 11 x 5 | tooth (C1, I2) abrasion, nasal bone comminuted fracture | mild | moderately severe | 0 | 115 |
| **50** | 13 | S | m | 36.3 |  |  | nasal bridge 3 x 3 | nasal bone tip fracture | mild | moderate | 0 | 30 |
| **54** | 14 | S | f | 34.8 |  |  | nasal bridge 1 x 1 | nasal bone tip fracture | mild | moderate | 0 | 30 |
| **59** | 15 | K | m | 20.4 | snout 1 x 0.5 |  | nasal bridge 1 x 1 | tooth (I1+2) abrasion, nasal bone tip fracture | mild | moderate | 5 | 35 |
| **60** | 16 | K | f | 35 | snout 1 x 1 | lip 1 x 0.5 |  |  | mild | mild | 30 | 35 |
| **72** | 17 | S | m | 31.2 |  |  | nasal bridge 2 x 2 | nasal bone tip fracture | mild | moderate | 0 | 30 |
| **78** | 18 | J | m | 37.4 | snout 1 x 0.5, 0.5 x 0.5, 1 x 0.5, nasal bridge 8 x 3, 2 x 2 | lip 1 x 0.5, gingiva 3 x 0.5 | gingiva 3 x 1, 2 x 2, nasal bridge 24 x 8, snout 2 x 2 | tooth (I1 + I2) fracture, nasal bone tip fracture, nasal bone comminuted fracture | severe | severe | 100 | 350 |
| **83** | 19 | K | f | 45.1 |  |  |  | tooth (C) tip fracture | - | - | 0 | 0 |
| **99** | 20 | S | m | 56.7 | snout 1 x 0.5 |  | nasal bridge circular 3 x 2 |  | mild | mild | 5 | 20 |
| **101** | 21 | K | m | 54.6 |  |  | nasal bridge 4 x 3, snout 2 x 2 | nasal bone tip fissure | mild | moderate | 0 | 45 |
| **102** | 21 | K | f | 51.2 |  |  | nasal bridge 4 x 2 | nasal bone tip fracture | mild | moderate | 0 | 50 |
| **103** | 22 | K | f | 32.2 |  |  | nasal bridge 4 x 2 | nasal bone tip fracture | mild | moderate | 0 | 50 |
| **109** | 23 | K | f | 20.9 |  |  | snout 1 x 1,  nasal bridge 4 x 2.5 | nasal bone tip fracture | mild | moderate | 0 | 65 |
| **111** | 24 | K | m | 65 | snout 1 x 1, 0.5 x 0.5, chin 2 x 3,  lip 1 x 2 | snout 2 x 1 | nasal bridge 0.3 x 0.3, snout 8 x 3 | tooth (I1 + I2) tip fracture | moderately severe | moderately severe | 50 | 80 |
| **113** | 25 | S | m | 6.6 |  |  | chin 3 x 1 |  | mild | mild | 0 | 15 |
| **125** | 25 | S | f | 6.8 |  |  | chin 2 x 1 |  | mild | mild | 0 | 15 |
| **127** | 26 | K | f | 35.4 |  | snout 2 x 0.5 | nasal bridge 2 x 3, snout 2 x 2 | tooth (I1) tip fracture, nasal bone tip fracture | moderate | moderately severe | 75 | 75 |
| **128** | 26 | K | f | 34.5 |  | snout 1.5 x 0.5, tongue 2 x 0.5 | nasal bridge 2 x 3, snout 5 x 2 | nasal bone tip fracture | moderate | moderately severe | 50 | 105 |
| **129** | 26 | K | f | 6.5 | carpus 2 x 1 |  | chin 3.5 x 2,  lower jaw 2 x 2 | mandibular symphysiolysis, unilateral mandibular fracture | moderate | severe | 5 | 205 |
| **130** | 26 | K | f | 5.7 |  |  | nasal bridge 1 x 1 |  | mild | mild | 0 | 15 |
| **133** | 26 | K | f | 5.2 | knee 3 x 2 |  |  |  | mild | mild | 5 | 5 |
| **136** | 27 | S | f | 43.4 |  |  | nasal bridge 4 x 3, snout 2 x 2 |  | mild | mild | 0 | 30 |

^a^ J = *JagerPro*, K = *Krefelder*, S = *Selfmade* ^b^ Sex: f = female, m = male
 ^c^ Carcass mass: Body mass without viscera, ID 1 = body mass without viscera and head
 ^d^ Lesion score according to ISO 10990-5, 1999: Animal (mammal) traps - Methods for testing restraining traps, Annex C; adjusted for wild boar trapping
 ^e^ Lesion score according to Proulx, Gilbert (2022): Mammal Trapping. Wildlife Management, Animal Welfare & International Standards: Alpha Wildlife Publications; adjusted for wild boar trapping
